# Characterization of Competitive ELISA and Formulated Alhydrogel Competitive ELISA (FAcE) for Direct Quantification of Active Ingredients in GMMA-Based Vaccines

**DOI:** 10.3390/mps3030062

**Published:** 2020-08-31

**Authors:** Omar Rossi, Maria Grazia Aruta, Alessandra Acquaviva, Francesca Mancini, Francesca Micoli, Francesca Necchi

**Affiliations:** GSK Vaccines Institute for Global Health s.r.l (GVGH), 53100 Siena, Italy; omar.x.rossi@gsk.com (O.R.); maria-grazia.x.aruta@gsk.com (M.G.A.); alessandra.x.acquaviva@gsk.com (A.A.); francesca.x.mancini@gsk.com (F.M.); francesca.x.micoli@gsk.com (F.M.)

**Keywords:** GMMA, vaccines, *Shigella*, Alhydrogel formulations, assay characterization, FAcE, cELISA

## Abstract

Generalized modules for membrane antigens (GMMA) represent a technology particularly attractive for designing affordable vaccines against Gram-negative bacteria. We explored such technology for the development of O-antigen-based vaccines against *Shigella* and nontyphoidal *Salmonella*. Adsorption of GMMA on Alhydrogel was required for abrogation of pyrogenicity in rabbits, and *Shigella sonnei* GMMA on Alhydrogel was well tolerated and immunogenic in humans. Quantification of key antigens in formulated vaccines was fundamental for release and to check stability overtime. Traditionally, the direct quantification of antigens adsorbed on aluminum salts has been challenging, and the quantification of each active ingredient in multicomponent formulated vaccines has been even more complicated. To directly quantify each active ingredient and unbound drug substances in formulated vaccines, we developed the Formulated Alhydrogel competitive ELISA (FAcE) and the competitive ELISA method, respectively. The methods were both fully characterized, assessing specificity, repeatability, intermediate precision, and accuracy, for *S. sonnei* OAg quantification, both in a single component or multicomponent GMMA formulation also containing *S. flexneri* GMMA. The developed immunological methods allowed us to fully characterize *Shigella* GMMA drug products, supporting their preclinical and clinical development. The same methods, already extended to GMMA from nontyphoidal *Salmonella* and *Neisseria meningitidis*, could be potentially extended to any antigen formulated on Alhydrogel.

## 1. Introduction

Generalized modules for membrane antigens (GMMA) are outer membrane exosomes shed from Gram-negative bacteria that are genetically modified to enhance the level of particle production [1]. Similar to native outer membrane vesicles, GMMA contain outer membrane lipids, outer membrane proteins, and soluble periplasmic components. They are self-adjuvanted particles, containing Toll-like receptor agonists, and present multiple bacterial antigens in their natural conformation as they faithfully resemble the composition of bacterial outer membrane. GMMA represent an attractive technology to develop high-yield and highly immunogenic particles, ideal for using as vaccines, especially to fight neglected diseases for which the balance between price and effectiveness is essential [2,3]. We have used GMMA technology to develop vaccines against enteric diseases, for example, *Shigella* and nontyphoidal *Salmonella* [3,4,5]. In both cases the O-antigen (OAg) portion of lipopolysaccharide (LPS) molecules was the key target for protective immunity. GMMA producing strains have been further manipulated to reduce the risk of inducing systemic reactogenicity, by playing with the lipid A acylation pattern [6,7]. The genetic manipulation of lipid A resulted in a reduction of *in vitro* pro-inflammatory response of over 500-fold as compared with wild-type lipid A [7]. Furthermore, the adsorption of GMMA with Alhydrogel allowed the abrogation of pyrogenicity in rabbits [5]. *S. sonnei* GMMA have been tested on Alhydrogel in humans, and the results have shown that they are well tolerated, immunogenic [8,9], and able to induce a strong anamnestic response upon boosting [10].

A panel of analytical techniques needs to be put in place to control each step of drug substance and drug product production. In particular it is essential to determine, with high specificity, the quality and quantity of each active ingredient present in the final formulation, as well as to follow over time their stability, as required by regulatory authorities. Traditionally, the direct quantification of antigens adsorbed on aluminium salts has been challenging, and antigens needed to be extracted using laborious and often ineffective desorption procedures [11,12,13,14,15]. Even more complicated is the quantification of each active ingredient of multicomponent formulated vaccines. To directly quantify the OAg in GMMA-based Alhydrogel-formulated vaccines, we have developed the Formulated Alhydrogel competive ELISA (FAcE) method [16]. Similarly, for the direct quantification of unbound drug substances in formulates, we developed a competitive ELISA (cELISA) assay.

Here, we present full development and characterization of the two methods in terms of accuracy, repeatability, reproducibility, specificity, and quantification range. In particular, we characterize cELISA and FAcE for *S. sonnei* OAg quantification, both when GMMA are in monovalent or multicomponent drug product formulations. FAcE assay on drug products and cELISA on drug product supernatants allow us to fully characterize the Alhydrogel-formulated vaccines, including lot-to-lot variations and the possibility of following their stability over time. The methods also work properly for multicomponent formulations and can be virtually extended to quantification of any Alhydrogel-formulated antigen.

## 2. Materials and Methods

### 2.1. GMMA Preparation (Drug Substance, DS)

*Shigella sonnei* 1790-GMMA [5], as well as GMMA from three different strains of *S. flexneri*, were produced by fermentation in a chemically defined media, purified by two tangential flow filtrations, and fully characterized using a panel of analytical methods, as previously reported [5].

Single component GMMA DS were diluted in PBS supplemented with Tween-20 (Sigma, St. Louis, MO, USA) 0.05% and 0.1% BSA (Sigma) at a nominal final concentration of 15 μg/mL (OAg based) prior to performing the assay.

A multicomponent GMMA DS mixture was obtained by mixing three different *S. flexneri* GMMA samples with *S. sonnei* GMMA in PBS Tween-20 0.05% and 0.1% BSA with a final nominal concentration of each sugar target of ~0.3 μg/mL (for *S. sonnei* OAg) and ~1 μg/mL (for each *S. flexneri* OAg).

### 2.2. Formulation of GMMA (Drug Product, DP)

*S. sonnei* 1790-GMMA were formulated at ~15.5 μg/mL or ~4 μg/mL nominal OAg content (used, respectively, for characterization of assay for single component and multicomponent DP) by mixing them for 2 h, at room temperature, with Alhydrogel 2% (Brenntag Biosector, Frederikssund, Denmark) (final concentration of 0.7 mg Al^3+^/mL) in Tris buffer (final concentration of 10 mM Tris, 9 g/L NaCl and pH 7.4).

GMMA purified from three different *S. flexneri* strains were also formulated (either as single component or as mixture of the three components) by mixing them for 2 h at room temperature (at ~26 μg/mL or ~15 μg/mL nominal OAg content of each active ingredient, respectively, for characterization of assay for single component as negative controls, and multicomponent DP) with Alhydrogel 2% (Brenntag Biosector, Denmark) (final concentration of 0.7 mg Al^3+^/mL) in water, and then conditioned with phosphate buffer (final concentration of 20 mM phosphate buffer, pH 6.5) and NaCl (final concentration 9 g/L).

The multicomponent formulation was obtained by bedside mixing the tri-component *S. flexneri* GMMA formulation (resulting in 15 μg/mL of each *S. flexneri* OAg) and *S. sonnei* single component drug product (resulting in ~4 μg/mL of *S. sonnei* OAg).

All formulations were characterized in terms of pH, osmolarity, and by visual inspection. Total proteins in DS used for formulations were quantified using the Lowry assay method [17] (in case of *S. sonnei*) or microBCA method (for *S. flexneri*), and non-adsorbed proteins in DP evaluated by SDS-PAGE Silver Staining analysis of the supernatant, obtained after centrifuging the formulation. GMMA adsorption on Alhydrogel was confirmed to be ≥95% for all single component and for the multicomponent formulations used here.

### 2.3. Working Principle of Assays

**Figure 1:**
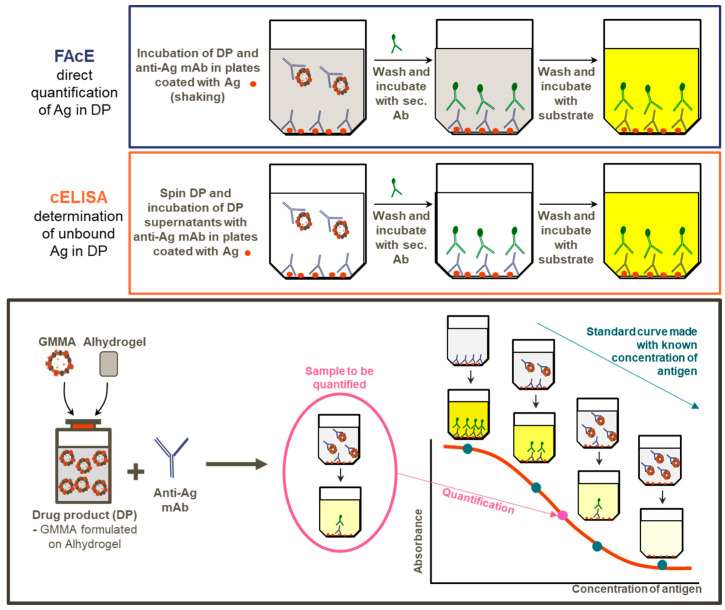
Visual descriptions of methods. Generalized modules for membrane antigens (GMMA) are formulated in Alhydrogel and their OAg content (active ingredient) can be directly quantified by Formulated Alhydrogel competitive ELISA (FAcE), whereas the quantity of unbound material can be determined by competititve ELISA (cELISA) performed on drug product supernatant. Both assays are quantitative and rely on standard curves made with a known quantity of antigen assayed in parallel to samples with unknown quantity. Samples and standard curve points compete with coated antigen for a limited amount of anti-antigen (anti-Ag) specific monoclonal antibodies (mAb) in a similar way.

Both cELISA for the direct quantification of *S. sonnei* OAg in DP supernatant and FAcE assay for the direct quantification of *S. sonnei* OAg in DP are based on the competition of an anti-*S. sonnei* LPS monoclonal antibody (mAb) for binding to the *S. sonnei* LPS antigen coated on the ELISA plate or the LPS of the GMMA suspension present in the ELISA wells. The OAg part of the LPS is the active ingredient of *Shigella* GMMA-based vaccines, and therefore we refer to this across the text. The more antigen that is present in the sample to be measured, the more mAb will bind to it, and less will be available to bind to the coated antigen. Consequently, the lowest signal is obtained with the highest quantity of measured antigen in the solution, and vice versa (Figure 1).

### 2.4. cELISA Protocol for Determination of Non-Absorbed S. sonnei GMMA in DP Supernatants

Our cELISA method was based on a reference standard curve built by 10 sequential dilutions (2.3-fold apart) of a freshly prepared *S. sonnei* GMMA solution, starting from a known concentration in terms of OAg of about 15 µg/mL, quantified through a combination of high performance anion exchange chromatography with pulsed amperometric detection (HPAEC-PAD) and NMR analysis that determine sugar content of the core region and OAg repeats to core ratio, respectively [5]. Two blank wells were also added containing dilution buffer only (PBS Tween 0.05% and 0.1% BSA). The test samples were GMMA solutions diluted in Tris buffer (final concentration of 10 mM Tris, 9 g/L NaCl, and pH 7.4) in the case of single components, or GMMA diluted in Tris buffer mixed 1:1 with phosphate NaCl buffer (20 mM phosphate buffer, pH 6.5, and NaCl 9 g/L) for multicomponent GMMA solutions; such samples containing GMMA in DP alum-free buffer mimic the potentially unbound GMMA in DP supernatants.

Five identical samples (A–E) were independently prepared by diluting the same solution containing GMMA (either for single component or for multicomponent assay characterization) (Figure S1A) in DP alum-free buffer. Each sample was assayed at 3 independent dilutions, each dispensed in 4 replicates within the same plate. The three independent dilutions were selected to fit within the most linear and central part of the standard curve, and were 0.25, 0.125, and 0.0625 μg/mL respectively. Samples not containing the *S. sonnei* OAg (samples N1, N2, and N3 constituted by 3 different *S. flexneri* GMMA solutions) were also assayed as negative controls to determine specificity of the assay at 0.25 μg/mL (the highest concentration assayed for samples containing *S. sonnei* OAg), in 4 replicates within the same plate.

The assay was run in independent triplicate in 96-well plates (Nunc round bottom Maxisorp ELISA plates), with the standard curve in duplicate on each plate. Plates were coated overnight at 4 °C with 100 μL/well of *Shigella sonnei* LPS at 0.5 µg/mL in PBS and blocked with 200 µL/well of 5% fat-free milk dissolved in PBS buffer at 25 °C for 1 h. Then, plates were washed three times with 250 µL of washing buffer (PBS with 0.05% Tween-20 (PBS-T)). Then, 800 μL of each independent dilution of test samples (and of positive and negative controls), as well as 400 μL of each dilution point of the standard curves, were spiked with an equal volume of anti-LPS mAb (Inbios International, Inc., Seattle, WA, USA, INB3192 purified mouse anti-*S. sonnei* IgG1) finally diluted 1:10,000 in PBS with 0.1% BSA and 0.05% Tween-20 in 2 mL Eppendorf tubes prior to be added into the test ELISA plates and immediately incubated for 2 h at 25 °C. Then, plates were washed three times with PBS-T. Next, 100 µL of secondary anti-mouse IgG conjugated to alkaline phosphatase (Sigma-Aldrich, A3438) were added to the plates and incubated for 1 h at 25 °C. After three more washes in PBS-T, 100 µL of p-nitrophenyl phosphate substrate solution (Sigma, N2770) were added and plates were incubated for 1 h at 25 °C. Absorbances at 405 and 490 nm were read and the difference between them (OD405nm–OD490nm) was calculated.

### 2.5. FAcE Protocol for Determination of S. sonnei OAg in DP

The standard curves were prepared by 10 sequential dilutions (2.3-fold apart) of Alhydrogel freshly formulated *S. sonnei* GMMA, starting from a known concentration in terms of OAg µg/mL (about 15.5 µg/mL), and two blanks (Alhydrogel diluent only). A standard curve was run in duplicate on each plate. The same freshly formulated sample used to build the standard curve was used as internal positive control (and therefore assayed as the other samples) to validate the whole plate within the assay. A plate was considered valid if the measured OAg concentration in the positive control was within a 70–130% accuracy range as compared with its nominal value. Test samples were represented by formulated GMMA (either as single component or as a multicomponent mixture). For each test sample, either five identical samples (A–E) in case of FAcE assay characterization for *S. sonnei* single component formulation, or four identical samples (sample A–D) in the case of FAcE characterization for quantification of *S. sonnei* OAg in *Shigella* multicomponent GMMA formulation, were independently prepared by diluting them in DP buffer (Figure S1B). Each sample was assayed at 3 independent dilutions, each dispensed in 4 replicates within the same plate. The three independent dilutions were selected to fit within the most linear (and central) part of the standard curve, and were 0.25, 0.125, and 0.0625 μg/mL, respectively. Samples not containing the *S. sonnei* OAg (samples N1, N2, and N3 constituted by 3 different *S. flexneri* single component formulates) were also assayed as negative controls to determine specificity of the assay at 0.25 μg/mL (the highest concentration assayed for samples containing *S. sonnei* OAg) in 4 replicates within the same plate.

The assay was run in independent triplicate 96-well plates (Nunc round bottom Maxisorp ELISA plates), with the standard curve in duplicate for each plate. Plates were coated overnight at 4 °C with 100 μL/well of *S. sonnei* LPS at 0.5 µg/mL and blocked with 200 µL/well of 5% fat-free milk dissolved in PBS buffer at 25 °C, for 1 h. Then, plates were washed three times with 250 µL of washing buffer (PBS with 0.05% Tween-20 (PBS-T)). Then, 800 μL of each independent dilution of test samples (and of positive and negative controls) and 400 μL of each dilution point of the standard curves were spiked with an equal volume of anti-LPS mAb (Inbios International, Inc. INB3192 purified mouse anti-*S. sonnei* IgG1), finally diluted 1:10,000 in PBS with 1% BSA and 0.05% Tween-20 in 2 mL Eppendorf tubes prior to be added in ELISA test plates, and immediately incubated for 2 h, at room temperature, shaking at 600 rpm using a MixMate (Eppendorf). Then, plates were washed three times with PBS-T and 100 µL of secondary anti-mouse IgG conjugated to alkaline phosphatase (Sigma-Aldrich, A3438) were added to the plates and incubated for 1 h, at 25 °C. After three more washes in PBS-T, 100 µL of p-nitrophenyl phosphate substrate solution (Sigma, N2770) were added, and plates were incubated for 1 h, at 25 °C. Absorbances at 405 and 490 nm were read and the difference between them (OD_405nm_−OD_490nm_) was calculated.

### 2.6. Calculations

Four parameter nonlinear (4PL) regression was applied to the standard curve and the amount of test samples were calculated from the equation Y = ((b/(a − Log_10_(X)))^(d) − 1)/c, where a, b, c, and d represents values of the curve parameters (respectively, being a = minimum asymptote, b = Hill’s slope, c = inflection point, and d = maximum asymptote), Y the amount determined, and X the absorbance reading. For the standard curve, the back calculation was performed by converting the observed readings of the standards to concentrations of the antigens using the 4PL equation described above. The OD values on which the 4PL curve was linear were automatically calculated and represented the quantification ranges of the standard curves.

Each plate was considered valid if the fitting of the standard curve presented R^2^ > 0.9, and if the OAg concentrations calculated from the internal positive control sample fell within 70–130% of its nominal concentration (in the case of FAcE assay). For each sample (test samples and positive control), OAg concentration was expressed as the average of all the values entering in the quantification range of the standard curve for each of the three plates assayed.

For precision determination, analysis was performed on three different days by two operators, each time in triplicate. Two different sets of experiments (for each assay) were performed to characterize the assays for monocomponent and multicomponent drug products.

Intermediate precision and repeatability were calculated using MiniTab software v.18 with Gage R & R protocol, considering as factors day and operator. For each experimental value obtained, the accuracy was also determined with the following formula: (experimental value/nominal value) * 100. To assess the overall accuracy for each assay, the average obtained from the differences of each experimental value from the nominal value was calculated with its confidence interval at 95% (CI 95%-indicated as percentage of nominal value).

## 3. Results

For both cELISA and FAcE, the following parameters were evaluated to characterize the assays: quantification range, precision, accuracy, and specificity. The parameters were evaluated both for single component and multicomponent preparations. Quantification of *S. sonnei* OAg was used for this purpose as the model antigen, because it was the active ingredient in the most advanced GMMA-based vaccine [5].

### 3.1. Quantification Range

The quantification range represents the range between the lowest and the highest amount of analyte in a sample which can be quantitatively determined with suitable precision and accuracy. Quantification range was calculated as the range between the minimum and maximum accepted OD (the linear part of the 4PL standard curve fitted to ODs). Results for each plate on each day and by each operator obtained for quantitation of single component formulations were considered to calculate the mean of the minimum and maximum OD and of the resulting minimum and maximum OAg (μg/mL)/well and corresponding standard deviations (Table 1). Both FAcE and cELISA showed a dynamic range ≥2 OD, resulting in a broad range of OAg (µg/mL)/well that could be directly determined within assays. Those ranges were 0.035–1.39 µg/mL and 0.015–1.12 µg/mL for FAcE and cELISA, respectively.

### 3.2. Accuracy

Accuracy is the measure of the closeness agreement between the nominal value of analyte measured (in our case the overall mean of the obtained values was considered to be the nominal value) and the values obtained within the assay. The average value of *S. sonnei* OAg measured by FAcE in DP was 17.47 and 4.95 µg/mL, respectively, for single component and multicomponent formulations, whereas the average of *S. sonnei* OAg measured in Alhydrogel-free DP buffer by cELISA were 1.67 and 0.32 µg/mL, respectively, for single component and multicomponent formulations. Accuracy and precision were not evaluated at multiple concentration levels, as the assay was performed by analyzing the sample at different dilutions and by averaging all the results fitting within the quantification range of the standard curves (the minimum and maximum accepted OD for each plate) obtained in each of the three plates. Table 2 and Table 3 (for cELISA and FAcE, respectively) report accuracy %, standard deviation, coefficient of variation %, standard error, and standard error % for each single OAg measurement performed (for test samples and controls, either when quantitating *S. sonnei* OAg in single or multicomponent drug products or drug product supernatants).

The results for both assays were accurate, either when OAg content was determined in single component or multicomponent drug products and drug product supernatants, respectively, for FAcE and cELISA. All measurements showed an accuracy with a maximum percentage deviation from the nominal value of 11.05% (considering a confidence interval at 95%) (Table 4).

### 3.3. Precision, Repeatability, and Reproducibility

Precision of the method expresses the closeness of agreement among multiple analyses of the same homogeneous representative sample tested under the prescribed conditions. Precision was considered at two levels, i.e., repeatability (intra-assay variation) and reproducibility (inter-assay variation). Repeatability measures the precision of the method under the same working conditions (same sample tested several times by the same operator in the same day, on the same lot of plates and using the same lot of critical reagents), and is due to those factors that have not been considered in the analysis (factors other than operator and day of analysis). Reproducibility is a measure of variability within laboratory variations. Results of repeatability and reproducibility are summarized in Table 4. Intermediate precision (CV% IP) estimates the assay reproducibility, hence, the variability in the same laboratory by performing the analysis in different days, by different operators, whereas repeatability (CV% R) measures the precision of the method under the same working conditions.

The results of both assays were reproducible (CV% IP < 10%) and repeatable (CV% R < 8%), with the majority of variance generally attributed to factors other than operator and day. In particular, cELISA for quantification of unbound material in drug product supernatants showed a good intermediate precision (CV% IP = 5.5% for single component and 8.2% for multicomponent) and good repeatability (CV% R = 4.3% for single component and 3.9% for multicomponent). In the case of assay performed on single components, the majority of variance was attributable to factors not considered in the analysis (% of variance component for repeatability = 60.86%) and, between the factors considered, only the operator was significant (*p* value = 0.008). In contrast, both day and operator were significant (*p* = 0.0001, the operator contributed to 35.42% of the variance and day contributed to 42.51% of the variance) in the case of quantification of *S. sonnei* OAg in multicomponent samples, and, for 22.7%, the variance was attributed to repeatability.

Similar to the observations for cELISA, in the case of the FAcE assay for direct quantification of *S. sonnei* OAg in monocomponent drug products, the majority of variance was also attributed to factors not considered in the analysis (% of variance component for repeatability = 64.21%) and between operator and day, only day was significant (*p* value = 0.008). For *S. sonnei* OAg quantification in multicomponent drug products, instead, the majority of variance was attributed to factors considered in the analysis (operator with 66.92% and day with 1.99% of contribution to variance components) with a contribution of repeatability equal to 31.09%; in this case contribution of operator was significant (*p* value = 0.0001).

### 3.4. Specificity

Another critical parameter of the assay is the ability to assess unequivocally the analyte in the presence of components which are expected to be present. In our case, other than the active ingredient represented by the *S. sonnei* OAg, the other GMMA components present in the formulations are lipids, proteins, lipoproteins, etc. Therefore, specificity was evaluated by assaying in FAcE and cELISA assays all the other single component drug products composing the multicomponent vaccines. The inability to determine the OAg concentration in all the wells assayed for those samples indicated the specificity of both FAcE and cELISA assays (Table 2 and Table 3). Moreover, the specificity of both assays was confirmed as the precision of both cELISA and FAcE remained good, also when other non-*S. sonnei* sugar components were present in the mixture (i.e., in assays characterized with multicomponent GMMA-based vaccines).

## 4. Discussion

GMMA is a promising technology for the development of affordable vaccines to fight neglected bacterial diseases causing millions of deaths every year. The most advanced GMMA-based vaccine, 1790-GAHB (*S. sonnei* GMMA formulated on Alhydrogel), has been tested in multiple clinical trials, and was well tolerated and immunogenic in European [8] and Kenyan [9] adults, as well as able to induce a strong anamnestic response after boosting [10]. Results with 1790-GAHB have pushed toward the development of a multicomponent GMMA-based vaccine, in which the *S. sonnei* component is formulated in Alhydrogel with GMMA from various *S. flexneri* strains, to cover the majority of the *Shigella* serotypes epidemiologically relevant worldwide. Similarly, a GMMA-based approach is under development and is entering clinical trials with the objective of producing a vaccine to fight invasive nontyphoidal *Salmonella* disease [3,4,6].

The direct and specific quantification of antigen content in Alhydrogel formulates, as well as the detection of unbound active ingredients in drug products, are essential for the full characterization of vaccines and represent a critical parameter to follow their stability over time. Using the FAcE method, the antigen/s of interest in Alhydrogel-formulated vaccines have been directly quantified [16] without the need to be extracted, and thus preventing possible antigen loss and alterations of critical epitopes. Similarly, by using the cELISA assay, the direct quantification of unbound drug substances in formulates have been effectively evaluated. This is critical to ensure that the quantity of unbound GMMA is below critical levels, as the presence of unbound GMMA over 10% in 1790GAHB, at the same dose used in humans, has been shown to induce pyrogenicity in rabbits [5]; thus, the possibility of quantifying with high sensitivity unbound GMMA in DP supernant is central to confirm that the drug product maintains a profile with low risk of inducing systemic reactogenicity.

In the present work, a full characterization of both methods has been described. *S. sonnei* OAg was selected as the model active ingredient and quantified both in single component and multicomponent DP formulations.

Our results demonstrate that FAcE and cELISA are extremely sensitive methods which are able to quantify up to 37 and 15 ng/mL of active ingredient, respectively, in a single component formulation, and up to 34 and 20 ng/mL of active ingredient, respectively, in a multicomponent formulation. They also showed good repeatability and high intermediate precision with CV% < 8% and 10%, respectively, and very good accuracy, represented by a maximum deviation of 11.05% from the expected value with a 95% CI. Importantly, good accuracy was maintained in both assays when multicomponent formulations were analyzed, demonstrating the lack of interference in more complex mixtures. Furthermore, both FAcE and cELISA results were highly specific, because they were unable to detect and quantify unrelated GMMA components from different *Shigella* serotypes. As further optimization and characterization steps for the cELISA method, spike and recovery assay could be considered to enforce accuracy. In contrast, for the FacE assay, the addition of a spike formulated on Alhydrogel could cause changes in the original formulation, therefore rendering a spike/recovery assay technically difficult.

The methods presented here support *S. sonnei* GMMA-based vaccine drug product release and should be critical for following its stability over time. Applications of the methods cover assessment of different quality aspects of vaccine characterization, such as efficiency of different formulation conditions, stability of formulated drug product, and lot-to-lot variations. Moreover, the possibility of characterizing specifically active ingredients in multicomponent vaccines is a crucial attribute in the modern vaccinology, as modern vaccines often contain multiple antigens in the same formulation, either to increase the broadness of vaccines to protect against multiple serotypes of the same pathogen, or to cover multiple diseases in one shot. For a direct quantification, FAcE is preferable to the O-phthalaldehyde assay (OPA) [15], direct Alhydrogel formulation immunoassay (DAFIA) [18], and flow cytometry [19] in terms of range of quantification, sensitivity, and specificity, with only the Luminex-based assay [20] showing a higher sensitivity, but a lower detection range. MicroBCA is suitable for direct quantification of Alhydrogel formulations, but it is not able to quantify specifically each active ingredient in complex vaccines mixtures.

cELISA is superior in terms of sensitivity and specificity as compared with other methods developed to obtain GMMA quantification in drug product supernatant such as micro-BCA or SDS-PAGE followed by silver staining. By using this assay, it is possible to quantify directly and specifically the active ingredients (e.g., *S. sonnei* OAg), whereas, for the other methods, the quantification is indirect. When tested in our lab, SDS-PAGE was characterized by lack of reproducibility and both micro-BCA and SDS-PAGE analysis had a sensitivity at least 10 times less than cELISA.

The same methods characterized here for *S. sonnei* GMMA candidate vaccine can be extended to GMMA from different *Shigella* serotypes (either as single component or in multicomponent formulations), as well as to other pathogens, as already verified in our lab for drug products containing *Salmonella* and *Neisseria meningitidis* GMMA. Furthermore, both assays have been verified to be applicable for detection of active ingredients of non-GMMA analytes formulated in Alhydrogel (i.e., protein antigens or classical polysaccharide-protein conjugates). The broad applicability of the methods makes them key analytical techniques to directly quantify active ingredients in complex Alhydrogel-formulated vaccines, supporting their preclinical and clinical development.

## Figures and Tables

**Table 1:** Quantification range.

Quantification Range			
		Mean All Repeats	Standard Deviation
**FAcE**	Maximum OD	2.7	0.20
	Min. OAg (µg/mL)/well	0.04	0.01
	Minimum OD	0.67	0.09
	Max. OAg (µg/mL)/well	1.39	0.23
**cELISA**	Maximum OD	2.69	0.13
	Min. OAg (µg/mL)/well	0.01	0.01
	Minimum OD	0.46	0.04
	Max. OAg (µg/mL)/well	1.12	0.24

**Table 2:** cELISA results for each single test performed. n.d., not detectable; St. Dev, standard deviation; SE, standard error; CV, coefficient of variation.

	Operator	Day	OAg (µg/mL)	St. Dev (µg/mL)	CV %	SE	SE %	Accuracy %
**Determination of *S. sonnei* OAg in single component DP supernatant**	A	1	1.60	0.19	11.67	0.031	1.945	95.97
	A	1	1.75	0.18	10.08	0.029	1.680	104.64
	A	1	1.76	0.22	12.46	0.037	2.077	105.35
	A	1	1.71	0.32	18.51	0.053	3.085	102.61
	A	1	1.66	0.31	18.66	0.052	3.111	99.49
	A	2	1.69	0.26	15.17	0.043	2.528	101.23
	A	2	1.74	0.12	7.03	0.020	1.172	104.22
	A	2	1.74	0.14	8.28	0.024	1.381	103.97
	A	2	1.82	0.36	19.66	0.060	3.277	108.76
	A	2	1.80	0.35	19.55	0.059	3.259	108.01
	A	3	1.58	0.18	11.63	0.031	1.939	94.63
	A	3	1.65	0.19	11.37	0.031	1.896	99.08
	A	3	1.72	0.15	8.47	0.024	1.412	103.31
	A	3	1.74	0.14	7.87	0.023	1.343	104.22
	A	3	1.64	0.30	18.10	0.050	3.016	98.48
	B	1	1.58	0.16	10.09	0.027	1.682	94.89
	B	1	1.67	0.17	10.36	0.029	1.726	100.11
	B	1	1.73	0.14	8.19	0.024	1.365	103.57
	B	1	1.73	0.31	18.02	0.052	3.003	103.91
	B	1	1.73	0.31	17.75	0.051	2.959	103.68
	B	2	1.44	0.22	15.03	0.036	2.506	86.26
	B	2	1.60	0.18	11.22	0.030	1.871	95.80
	B	2	1.64	0.18	11.18	0.031	1.863	98.19
	B	2	1.71	0.33	19.19	0.055	3.198	102.22
	B	2	1.71	0.33	19.32	0.055	3.219	102.36
	B	3	1.62	0.23	14.39	0.039	2.399	96.82
	B	3	1.61	0.13	8.09	0.022	1.348	96.41
	B	3	1.58	0.15	9.35	0.025	1.558	94.88
	B	3	1.57	0.30	19.15	0.050	3.192	94.03
	B	3	1.55	0.30	19.64	0.051	3.274	92.91
**Determination of *S. sonnei* OAg on multicomponent DP supernatant**	A	1	0.35	0.05	14.29	0.008	2.382	107.84
	A	1	0.35	0.04	10.89	0.006	1.815	108.80
	A	1	0.35	0.03	7.28	0.004	1.248	109.72
	A	1	0.34	0.03	8.47	0.005	1.474	106.41
	A	1	0.34	0.04	12.37	0.008	2.337	104.84
	A	2	0.35	0.02	6.49	0.004	1.082	108.61
	A	2	0.36	0.03	7.06	0.004	1.176	112.31
	A	2	0.35	0.03	7.49	0.004	1.285	108.89
	A	2	0.35	0.02	6.36	0.004	1.107	108.55
	A	2	0.34	0.02	6.00	0.004	1.134	106.06
	A	3	0.30	0.03	10.61	0.005	1.768	93.29
	A	3	0.31	0.02	7.05	0.004	1.176	95.22
	A	3	0.30	0.03	9.73	0.005	1.669	94.26
	A	3	0.30	0.03	10.97	0.006	1.910	93.36
	A	3	0.30	0.03	8.71	0.005	1.646	94.89
	B	1	0.30	0.03	11.11	0.006	1.851	94.59
	B	1	0.31	0.04	13.52	0.007	2.253	97.28
	B	1	0.31	0.04	12.16	0.006	2.085	96.93
	B	1	0.31	0.10	30.77	0.017	5.356	97.69
	B	1	0.31	0.03	8.94	0.005	1.690	96.16
	B	2	0.31	0.02	5.92	0.003	0.986	97.23
	B	2	0.33	0.02	6.38	0.003	1.063	101.45
	B	2	0.32	0.02	7.52	0.004	1.289	100.11
	B	2	0.32	0.09	28.60	0.016	4.979	98.95
	B	2	0.32	0.03	8.95	0.005	1.692	99.90
	B	3	0.34	0.05	14.19	0.008	2.365	105.10
	B	3	0.28	0.03	10.03	0.005	1.672	88.59
	B	3	0.29	0.05	18.89	0.009	3.240	89.02
	B	3	0.30	0.09	31.41	0.016	5.468	93.48
	B	3	0.29	0.04	14.58	0.008	2.756	90.47
**N1**	A	1	n.d	---	---	---	---	---
	A	2	n.d	---	---	---	---	---
	A	3	n.d	---	---	---	---	---
	B	1	n.d	---	---	---	---	---
	B	2	n.d	---	---	---	---	---
	B	3	n.d	---	---	---	---	---
**N2**	A	1	n.d	---	---	---	---	---
	A	2	n.d	---	---	---	---	---
	A	3	n.d	---	---	---	---	---
	B	1	n.d	---	---	---	---	---
	B	2	n.d	---	---	---	---	---
	B	3	n.d	---	---	---	---	---
**N3**	A	1	n.d	---	---	---	---	---
	A	2	n.d	---	---	---	---	---
	A	3	n.d	---	---	---	---	---
	B	1	n.d	---	---	---	---	---
	B	2	n.d	---	---	---	---	---
	B	3	n.d	---	---	---	---	---

**Table 3:** FAcE assay results for each single test performed. n.d., not detectable; St. Dev, standard deviation; SE, standard error; CV, coefficient of variation.

	Operator	Day	OAg (µg/mL]	St. Dev (µg/mL)	CV %	SE	SE %	Accuracy %
**Determination of *S. sonnei* OAg in single component DP supernatant**	A	1	17.73	2.98	16.82	0.497	2.803	101.44
	A	1	14.77	1.84	12.48	0.307	2.079	84.50
	A	1	15.71	1.88	11.94	0.322	2.047	89.91
	A	1	17.88	2.08	11.61	0.361	2.021	102.30
	A	1	15.77	3.96	25.10	0.748	4.743	90.23
	A	2	16.83	1.40	8.30	0.233	1.383	96.32
	A	2	18.23	4.12	22.59	0.740	4.058	104.32
	A	2	19.04	1.93	10.15	0.322	1.691	108.95
	A	2	21.36	3.14	14.70	0.523	2.449	122.24
	A	2	18.56	2.89	15.54	0.481	2.591	106.22
	A	3	17.59	2.05	11.64	0.362	2.059	100.68
	A	3	17.74	3.22	18.14	0.569	3.206	101.52
	A	3	19.03	1.91	10.06	0.338	1.778	108.88
	A	3	18.47	2.69	14.58	0.476	2.578	105.69
	A	3	18.74	2.16	11.54	0.382	2.040	107.27
	B	1	15.46	2.54	16.43	0.423	2.738	88.46
	B	1	17.09	1.62	9.50	0.271	1.584	97.78
	B	1	17.85	2.34	13.13	0.390	2.188	102.12
	B	1	17.25	2.30	13.32	0.388	2.251	98.72
	B	1	18.47	3.13	16.94	0.521	2.823	105.68
	B	2	17.25	3.49	20.21	0.581	3.369	98.69
	B	2	19.90	3.00	15.06	0.499	2.509	113.89
	B	2	19.09	3.25	17.03	0.542	2.838	109.24
	B	2	18.67	2.46	13.15	0.415	2.223	106.81
	B	2	18.02	1.71	9.50	0.289	1.605	103.11
	B	3	17.02	3.87	22.76	0.732	4.302	97.39
	B	3	15.05	2.33	15.48	0.440	2.925	86.15
	B	3	15.47	2.14	13.80	0.404	2.608	88.55
	B	3	15.40	2.44	15.85	0.470	3.051	88.15
	B	3	14.82	1.72	11.62	0.331	2.236	84.79
**Determination of *S. sonnei* OAg on multicomponent DP supernatant**	A	1	4.62	0.54	11.74	0.090	1.957	93.39
	A	1	4.55	0.54	11.80	0.092	2.023	91.89
	A	1	4.70	0.36	7.55	0.062	1.315	95.05
	A	1	4.61	0.51	11.07	0.096	2.091	93.28
	A	1	4.57	0.51	11.09	0.084	1.848	92.29
	A	2	4.77	0.73	15.24	0.125	2.613	96.51
	A	2	4.62	0.65	14.12	0.114	2.457	93.47
	A	2	4.70	0.61	13.05	0.116	2.466	94.97
	A	2	4.73	0.58	12.29	0.097	2.048	95.56
	A	2	4.75	0.48	10.03	0.082	1.721	95.99
	A	3	4.85	0.70	14.42	0.122	2.509	98.06
	A	3	5.05	0.73	14.40	0.138	2.721	102.19
	A	3	5.47	0.49	8.87	0.081	1.478	110.67
	A	3	5.46	0.61	11.16	0.105	1.914	110.47
	A	3	5.46	0.73	13.28	0.126	2.312	110.42
	B	1	5.25	0.65	12.30	0.122	2.325	106.04
	B	1	5.46	0.67	12.29	0.112	2.049	110.45
	B	1	5.41	0.81	15.04	0.140	2.579	109.43
	B	1	5.22	0.78	14.94	0.136	2.600	105.52
	B	1	5.15	0.77	15.04	0.146	2.842	104.14
	B	2	4.84	0.33	6.83	0.055	1.138	97.75
	B	2	4.98	0.47	9.50	0.081	1.630	100.67
	B	2	4.69	0.53	11.24	0.092	1.956	94.72
	B	2	4.80	0.52	10.82	0.098	2.045	97.09
	B	2	12.51	1.16	9.27	0.193	1.544	108.52
	B	3	11.50	1.02	8.88	0.170	1.480	99.79
	B	3	10.60	1.48	14.00	0.247	2.333	91.98
	B	3	12.4	1.18	9.49	0.196	1.582	107.60
	B	3	11.16	1.86	16.66	0.310	2.777	96.84
	B	3	10.98	1.07	9.73	0.178	1.622	95.28
**Positive control (single component formulations)**	A	1	12.51	1.16	9,.27	0.193	1.54	108.52
	A	2	11.5	1.02	8.88	0.170	1.48	99.79
	A	3	10.6	1.48	14.00	0.247	2.33	91.98
	B	1	12.4	1.18	9.49	0.196	1.58	107.60
	B	2	11.16	1.86	16.66	0.310	2.77	96.84
	B	3	10.98	1.07	9.73	0.178	1.62	95.28
**N1**	A	1	n.d	---	---	---	---	---
	A	2	n.d	---	---	---	---	---
	A	3	n.d	---	---	---	---	---
	B	1	n.d	---	---	---	---	---
	B	2	n.d	---	---	---	---	---
	B	3	n.d	---	---	---	---	---
**N2**	A	1	n.d	---	---	---	---	---
	A	2	n.d	---	---	---	---	---
	A	3	n.d	---	---	---	---	---
	B	1	n.d	---	---	---	---	---
	B	2	n.d	---	---	---	---	---
	B	3	n.d	---	---	---	---	---
**N3**	A	1	n.d	---	---	---	---	---
	A	2	n.d	---	---	---	---	---
	A	3	n.d	---	---	---	---	---
	B	1	n.d	---	---	---	---	---
	B	2	n.d	---	---	---	---	---
	B	3	n.d	---	---	---	---	---

**Table 4:** Evaluation of accuracy and precision for FAcE and cELISA. CI, confidence interval; CV, coefficient of variation.

		CI 95% (Deviation from the Nominal Value %)	CV% IP (Intermediate Precision)	CV% R (Repeatability)	CV% Operator	CV% Day
**FAcE**	Single component	6.52–11.05	9.83	7.88	2.03	5.52
	Multicomponent	5.18–7.62	8.11	4.52	6.63	1.14
**cELISA**	Single component	3.31–6.16	5.53	4.31	3	1.71
	Multicomponent	5.49–7.88	8.23	3.87	4.9	5.37

## Supplementary Materials

The following are available online at https://www.mdpi.com/2409-9279/3/3/62/s1, Figure S1: Plates layout. (A) Plates layout for cELISA (to characterize assay both for single and multicomponent) and for FAcE characterization (single components); (B) Plates layout for FAcE (to characterize assay for multiple components).
