# Supplementary figures and images for: Characterization of Competitive ELISA and Formulated Alhydrogel Competitive ELISA (FAcE) for Direct Quantification of Active Ingredients in GMMA-Based Vaccines
Source: Methods Protoc. 2020 Aug 31;3(3):62. doi: 10.3390/mps3030062 (PMC7563494; doi:10.3390/mps3030062)

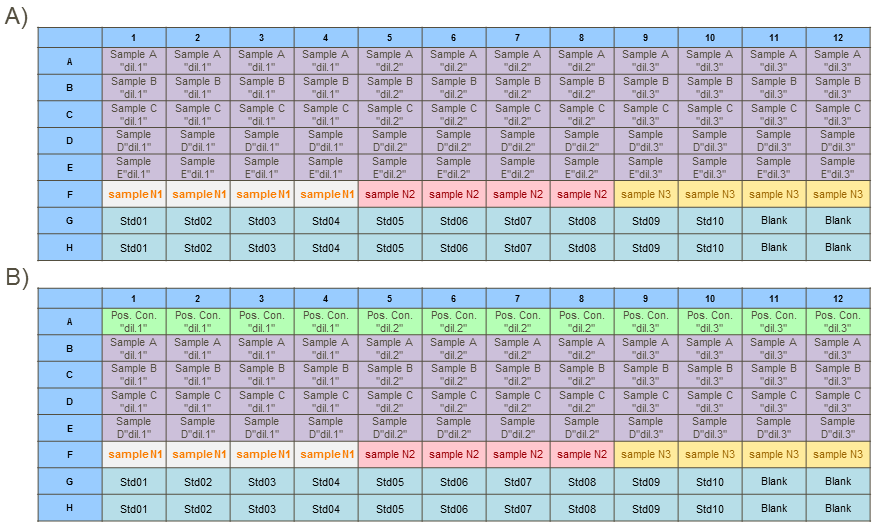

Supplement: Supplementary file 1 [file mps-03-00062-s001.zip › Slide2.tif]
